# Compromise or Capitulation? US Food and Drug Administration Jurisdiction Over Tobacco Products

**DOI:** 10.1371/journal.pmed.1000118

**Published:** 2009-07-28

**Authors:** Stanton A. Glantz, Richard Barnes, Sharon Y. Eubanks

**Affiliations:** 1 Center for Tobacco Control Research and Education, University of California San Francisco, San Francisco, California, United States of America; 2 Attorney in private practice, McLean, Virginia, United States of America

## Abstract

Stanton Glantz and colleagues critique the recent policy decision in the United States to grant the FDA regulatory authority over tobacco products, a decision that has broad but not unanimous support among health care professionals.

**Summary Points**

- On June 12, 2009, the US Congress passed compromise legislation granting the Food and Drug Administration (FDA) jurisdiction over tobacco products, repealing federal pre-emption of state and local regulation of tobacco advertising, and requiring graphical warning labels on tobacco products.
- Past legislative compromises with the tobacco industry have led to short-term public health gains at the expense of long-term progress.
- The law offers the tobacco industry an opportunity to rehabilitate its image and products because they are now “FDA regulated.”
- Tobacco interests that have violated US racketeering law are inappropriately represented on the Scientific Advisory Committee that influences FDA regulations.
- The challenge for the compromise law's advocates will be to accept responsibility for these problems and see that their negative consequences do not materialize.

On June 12, 2009, the United States Congress passed the Family Smoking Prevention and Tobacco Control Act (S. 982), granting the US Food and Drug Administration (FDA) authority regulating tobacco products. The *idea* of FDA regulation of tobacco has broad [1], but not unanimous, support among medical and public health professionals. This law has many strengths (Table 1): it grants the FDA general authority over tobacco products, including the ability to reduce (but not eliminate) nicotine, requires improved warning labels on cigarette and other tobacco packages, and implements the rules limiting marketing of tobacco products to youth that the FDA issued in 1996. It also repeals pre-emption of state and local regulation of tobacco marketing and advertising, and grants states and localities broad rights to regulate or prohibit the sale, distribution, possession, exposure to, access to, or use of tobacco products.

**Table 1:** Major provisions of FDA law (S. 982, June 12, 2009).

Positive	Negative
Gives Secretary of Health and Human Services authority to issue regulations regarding existing tobacco products and premarketing approval of new tobacco products and assigns regulatory authority to the FDA (§101)	Includes strong federal pre-emption (except fire safety standards) provisions of state and local governments with respect to product standards, premarket approval, adulteration, misbranding, labeling registration, and good manufacturing standards (§916(a))
	Grandfathers from premarketing approval all tobacco products commercially marketed as of February 15, 2007 (§905(j)) and some products introduced within 21 months of law taking effect (§910(a)(2)(B))
Gives the Secretary authority to impose restrictions on advertising and promotion of tobacco products (§906(d)(1))	
	Prohibits requiring a prescription for tobacco products (§906(d)(1))
Requires the Secretary to issue regulations on the promotion, marketing, sale, and distribution of tobacco products by means other than a direct, face-to-face exchange between a retailer and a consumer (§906(d)(4))	
Empowers Secretary to issue tobacco product standards for all tobacco products (§907)	
Bans use of many flavoring agents (e.g., strawberry, chocolate, coffee) (§907(a)(1)(A))	Excludes menthol^a^ (§907(a)1A)
Empowers Secretary to require the reduction or elimination of tobacco constituents and harmful components of tobacco products (§907(a)(4))	Prohibits requiring reduction of nicotine to zero (§907(d)(3)(B))
	Requires Secretary to consider in promulgating regulations “technical achievability” (§907(b)(1), 907(d)(2)), the possibility of “the creation of a significant demand for contraband” (§907(b)(2)), and choosing an effective date “to minimize, consistent with public health, economic loss to, and disruption of or dislocation of, domestic and international trade” (§907(d)(2))
	Prohibits banning all cigarettes, smokeless products, cigars, etc. (§907(d)(3)(A))
	Requires Secretary of Health to invite Secretary of Agriculture to provide information and analysis regarding any proposed tobacco product standard (§907(c)(2)(D)), even though Secretary of Health is prohibited from issuing regulations regarding growing, cultivation, or curing of raw tobacco (§4)
Repeals current federal pre-emption of state and local authority to issue rules regulating marketing and advertising, and grants to states broad rights to regulate or prohibit the sale, distribution, possession, exposure to, access to, or use of tobacco products (§916(a)(1), 203)	Pre-empts state and local regulation of product, except as it relates to fire safety (§916(a)(1))
Creates 12-member Tobacco Products Scientific Advisory Committee, which plays a central role in reviewing all proposed scientific findings and regulations (§917(a))	Requires 3 nonvoting members be representatives of tobacco interests (2 manufacturers and 1 grower) (§917(b)(1)(A)(iv–vi)); limits conflict-of-interest disqualification to only 18 months prior to appointment to the Committee (§917(b)(1)(C))
Establishes user fees paid by tobacco companies to fund regulatory activities (§919)	No adjustment for inflation after 2019 (§919(b)(1)(K)), potentially limiting resources for future regulation
Implements the restrictions on marketing to youth the FDA issued in 1996 (§102(a))	
Prohibits free sampling of smokeless tobacco at football, basketball, baseball, soccer, or hockey events (§102(d)(3)(B))	Allows free sampling at other venues, including rodeos and stock car races^a^ (§102(d)(3)(B))
Rules issued on sales to minors exempt from Congressional review under Administrative Procedures Act (§102(a)(7))	All other rules are subject to Congressional review
Manufacturers may not make statements through the advertising or media directed to consumers “that could result in consumers believing that the product is endorsed for use by the Food and Drug Administration or in consumers being misled about the harmfulness of the product because of such regulation, inspection, or compliance”^b^ (§103(tt))	The Supreme Court [42] has found that commercial speech may be regulated by the government only if it is false or misleading; First Amendment protection may be afforded to statements of the existence of FDA regulation, based on this precedent
Mandates large graphical warnings on the front and back of packages (§201, 204)	

a These provisions can be strengthened through the rule-making process.

b Asserting the right to use the fact that their products are regulated by the FDA in advertising may have been the reason that Michael Szymanczyk, Chairman and CEO of Altria (Philip Morris USA's parent company) wrote, in his letter endorsing the Family Smoking Prevention and Tobacco Control Act, “we have repeatedly expressed First Amendment reservations about certain provisions, including those that could restrict a manufacturer's ability to truthfully communicate about tobacco products” [43].

The new FDA law is a compromise between tobacco control organizations and the Philip Morris tobacco company that has its origins in the failed 1997 “global settlement” negotiated to settle litigation against the tobacco industry [2],[3]. These organizations and their Congressional allies believed that the only way to win regulatory authority over tobacco products was to compromise with the tobacco industry. Some of the compromises, such as prohibiting the FDA from eliminating all nicotine from tobacco products, requiring a prescription for tobacco products, or banning them outright (Table 1) do not create overly burdensome regulatory hurdles or fundamental long-term advantages or legitimacy for the industry. Other provisions, however, do.

## Lessons from Past Compromises with the Tobacco Industry

The FDA Act is not the first time that public health advocates have compromised with tobacco companies to pass legislation. History demonstrates that the tobacco companies think in the long term and, when forced, will accept short-term setbacks to protect long-term interests.

The first federal health legislation addressing tobacco was the Federal Cigarette Labeling and Advertising Act, passed in 1965. This law, passed in the wake of growing public concern triggered by the original 1964 Surgeon General's report that concluded that smoking caused lung cancer (in men), which accelerated calls for restrictions on cigarette marketing at all levels of government around the country ([4], p. 205), mandated now-familiar (and worn-out) “Surgeon General's Warnings” on the side of cigarette packages and, later, in cigarette advertising.

While this legislation was viewed as a step forward, it was the tobacco companies who emerged as the long-run winners. The price of the law was federal pre-emption of any controls on cigarette advertising ([5], pp. 254–257), including at the state or local level. The result was to choke off all subsequent local efforts that had anything to do with cigarette advertising. The warning labels also provided the tobacco companies an important defense in court to fight liability for the disease and death that their products cause. At the time the FDA bill passed—44 years later—the US still had the weakest, least effective warning labels in the world [6]. Indeed, one of the arguments for the FDA bill was that it required graphical warning labels and repealed this pre-emption [7].

The next compromise came in 1970, when health advocates worked with tobacco companies to remove cigarette advertising from television and radio. This compromise, too, turned out to be a strategic error because once the pro-tobacco advertisements were gone, the Federal Communications Commission “Fairness Doctrine” that had required free anti-smoking messages to counter the tobacco advertisements no longer applied. These anti-smoking advertisements were having a more profound effect on consumers than the cigarette advertisements and the industry wanted them gone [8]. The tobacco companies simply shifted their marketing to other venues and public health messages disappeared from the airwaves.

The one unequivocal public health success won against tobacco in Congress was legislation making airline flights smoke-free (in 1988 for flights of two hours or less, in 1989 for flights of six hours or less, and in 2000 for all flights to and from the US), despite vehement opposition from the tobacco industry ([5], pp. 303–306). Not only did these laws improve the health and comfort of the flying public and flight attendants for whom airliners were workplaces, but they contributed to the declining social acceptability of smoking, which reduces smoking.

## The New FDA Law

There are many elements of this new legislation that could prove problematic in achieving effective control of tobacco products and their manufacturers (Table 1). The law imposes strong pre-emption (except fire safety standards) of state and local governments with respect to product standards, premarket approval, adulteration, misbranding, labeling registration, and good manufacturing standards. Pre-emption is a key tobacco industry strategy for preventing innovation and development of strong tobacco control policies [9]. Indeed, it was the lack of such pre-emption that permitted states to develop and implement fire safety standards for cigarettes while the industry effectively blocked federal action. This pre-emption could block the emerging movement to regulate cigarette butts as toxic waste [10].

While nominally giving the Secretary broad authority to regulate tobacco products and their promotion, the law establishes procedures that will make it difficult to issue meaningful regulations. Rather than an unequivocal charge to protect the public health, the regulations must consider, among other things, “technical achievability” and the possibility of “creating a significant demand for contraband” (Table 1). These requirements provide fertile ground for years of lawsuits against any regulation the industry opposes [11].

Evidence in millions of pages of tobacco industry documents now publicly available demonstrates that Philip Morris has been planning for FDA legislation for well over a decade and illustrates how such regulation fits into its business plan [12],[13]. Even a perfect (from a public health perspective) law would benefit Philip Morris because it will tend to perpetuate the status quo and Philip Morris is the market leader. Philip Morris's apparent reasons for supporting FDA regulation, however, extend well beyond this fact. In 1998, a Philip Morris legal white paper, “Design, Manufacturing and Marketing of Tobacco Products: Towards a Sensible Regulatory Framework,” explained that “reasonable” FDA authority “would continue to permit adults to assume the *inherent* risks of smoking, while allocating to the government the twin tasks of ensuring that manufacturers don't create *additional* risk through their design and manufacturing processes, on the one hand, and continuing to conduct appropriate research about the nature of the inherent risks and keeping consumers *informed* of them on the other” [emphasis in original] [14]. Like warning labels, this white paper appears to suggest that Philip Morris anticipated the legislation would help shift liability from cigarette companies for the dangers of smoking and place responsibility for informing consumers of the risk upon the government.

In 1996, Philip Morris implemented Project Sunrise, an 11-year-plan to restore its reputation as a “responsible company,” to maintain its political influence (the key to survival for any tobacco company), to recruit quality staff, and to maintain its customer base [13]. Publicizing the death and destruction the tobacco industry causes has been a staple of effective tobacco control media campaigns, beginning in California [15] and used to strong effect in the American Legacy Foundation's “truth” campaign [16],[17],[18]. Indeed, distrust of tobacco companies is a powerful message to prevent smoking and to stimulate cessation attempts [19],[20].

A particularly problematic aspect of the law is the Tobacco Products Scientific Advisory Committee that is required to include nonvoting members from the tobacco manufacturers and growers. The Committee is charged with advising the Secretary of Health and Human Services on, among other things, questions regarding nicotine dependence, whether menthol (present in about 70% of cigarettes smoked by African Americans [21]) should be removed from tobacco products, and on virtually all regulations and health issues relating to tobacco products. This committee will play an important role in adjudicating complex and controversial scientific issues about whether meaningful “harm reduction” is possible with tobacco products through product modification at a time that there is little to no scientific literature on the actual links between a variety of tobacco-related diseases and specific ingredients. Before promulgating any regulation, the Secretary is required to afford the Committee an opportunity to submit recommendations with respect to the proposed regulations.

While industry representatives participate as nonvoting members of other FDA scientific advisory committees, there is significant negative historical precedent for the effects of including tobacco industry representatives in this committee. In 1968 the National Cancer Institute created the Tobacco Working Group (TWG), which included health experts and industry representatives, to advise its Smoking and Health Program ([22], pp. 155–162). Federal District Judge Gladys Kessler summarized how the industry used its TWG membership in the Group in her 2006 ruling that Philip Morris and other elements of the tobacco industry violated the federal Racketeer Influenced and Corrupt Organization (RICO) Act: “Participation by industry representatives proved valuable [to the industry] by allowing Defendants to keep abreast of what the United States Government was doing with respect to smoking and health issues. Their participation also provided a mechanism by which defendants could try to influence the United States Government's activities in the smoking and health area…The [racketeering] Enterprise engaged in a concerted effort to prevent, curtail, and ultimately to neutralize the TWG's efforts to evaluate cigarettes' [health] effects using an animal bioassay…” ([23], ¶334, 337). This industry is not known for its honesty in the scientific arena [24]–[37] and, accordingly, has no legitimate place as inside participants in FDA rule-making.

Notably, while the law's “findings” quote Judge Kessler's conclusions on the industry's marketing to youth, it is silent on her extensive findings on the industry's corruption of science ([23], ¶3864, 3871, 3877, 3897, 3907, 3731). In particular, Judge Kessler found that not only had the industry engaged in these illegal practices in the past, but that they were continuing to do so and likely to continue to do so in the future ([23], pp. 1601–1612).

On May 22, 2009, after the Senate Health Committee finished its work on the bill but before the vote in the full Senate, the District of Columbia Court of Appeals unanimously upheld Judge Kessler's ruling in strong terms: “Defendants knew of their falsity at the time and made the statements with intent to deceive. Thus, we are not dealing with accidental falsehoods or sincere attempts to persuade; defendants' liability rests on deceits perpetrated with knowledge of their falsity” ([38], p. 42). These findings by an impartial judiciary (and the evidence they are based on) justify excluding industry representatives from serving on this committee as participants *inside* the FDA rule-making process.

Including tobacco growers on the committee—and the requirement that the Secretary of Health and Human Services consult with the Secretary of Agriculture on proposed rules—is even stranger. The proposed law specifically excludes regulation of tobacco leaf not in the possession of a manufacturer of tobacco products. Clearly, these provisions have no rational basis to the stated intent of the legislation or the mission of the FDA.

## Implications for Global Tobacco Control

Including the tobacco industry in this committee also violates Article 5.3 of the World Health Organization Framework Convention on Tobacco Control (FCTC) [39], the first global public health treaty, which has been ratified by 164 nations. (The US has signed, but not yet ratified the treaty.) In recognition of the tobacco industry's corrosive influence on government, FCTC Article 5.3 commits parties to protecting tobacco control policy and development from the tobacco industry. The implementing guidelines for FCTC Article 5.3 are unequivocal: “Parties should not allow any person employed by the tobacco industry or any entity working to further its interests to be a member of any government body, committee or advisory group that sets or implements tobacco control or public health policy” ([40], §23.4.8).

Ironically, many of the US groups that pushed the current FDA bill also worked hard to win a strong FCTC and are urging US ratification of the treaty [39]. The multinational tobacco companies will almost certainly use the precedent in the FDA bill to undermine implementation of the FCTC elsewhere, particularly since leading health advocates in the United States have been publicly defending this provision. Even though the US is not yet a party to the FCTC, US advocates must consider the global public health impacts of their actions here.

## Recognizing Changed Circumstances

Finally, the basic terms of the bill were negotiated several years ago, in a wholly different political climate. At that time, it appeared that Republicans would dominate government well into the future. The 2008 election represented a sea change in American politics, including a President who has repeatedly emphasized his commitment to respecting science, depoliticizing the role of science in government decision-making, and rejoining the international community. This FDA legislation does not acknowledge this changed political climate or recognize the opportunity it created to accomplish more than when the bill was originally negotiated. Advocates for the bill also did not press the implications of the strong Court of Appeals decision upholding Judge Kessler's ruling that the tobacco companies were racketeers to remove industry representatives from the Scientific Advisory Committee.

In July 1963, six months before the 1964 Surgeon General's report was released ([22], pp. 50–52), the Director of Public Relations at Brown and Williamson Tobacco wrote his colleagues that “The consensus is that the industry is in a ‘grave crisis,’ and the philosophy is ‘to expect the worst and work for the best.’ Of course, the greatest cause for alarm is the forthcoming Surgeon General's report, which is expected to be detrimental to the industry… because indications point to a strong indictment of tobacco, with possible ‘root shaking’ consequences…[There are] feelings of frustration, inadequacy, and fear that exist among those engaged in representing the tobacco industry…” [41].

Rather than mustering the political will to realize the vulnerable industry's worst fears, Congress enacted the 1965 Federal Cigarette Labeling and Advertising Act, which protected the industry for half a century. It could be an even greater mistake to have missed the opportunity to enact FDA regulation of tobacco products that actually reins in the industry's largest player rather than enacting an insurance policy for the industry's future. We, after all, have insight into what Philip Morris wants from this bill and how it may serve their long-term interests in maximizing sales of cigarettes and other tobacco products [12],[13].

Smoke-free airlines show that a united public health community, working with Congress, can beat the tobacco industry. The repeal of pre-emption of state and local regulation of tobacco advertising and the requirement for graphical health warnings represents a clear step forward. The public health community had built exceptionally strong public support for the *idea* of FDA regulation of tobacco. A pro-health president, committed to legitimate science and re-entering the global community, and strong findings from the courts that the tobacco industry represents an ongoing racketeering enterprise should have helped. 

The effectiveness with which the FDA will be able to regulate tobacco products, however, remains in doubt and the law offers the tobacco industry an opportunity to rehabilitate its image and products because they are now “FDA regulated.” Most concerning, the precedent of the United States enacting a law that violates Article 5.3 of the FCTC ([40], §23.4.8), with active support of major public health groups and pro-health Congressional leaders, could undermine international tobacco control efforts.

The challenge for the compromise law's advocates will be to accept responsibility for these problems and work to ensure that their negative consequences do not materialize.

## Abbreviations

FCTC: Framework Convention on Tobacco Control
FDA: Food and Drug Administration
TWG: Tobacco Working Group

## References

[1] Yang JS, Novotny TE (2009). Policy coherence in US tobacco control: Beyond FDA regulation.. PLoS Med.

[2] Givel M, Glantz SA (2004). The “global settlement” with the tobacco industry: 6 years later.. Am J Public Health.

[3] Fox BJ, Lightwood JM, Glantz SA (1998). A public health analysis of the proposed resolution of [the 1997 United States] tobacco litigation.. http://repositories.cdlib.org/ctcre/tcpmus/US1998.

[4] Wagner S (1971). Cigarette country; Tobacco in American history and politics.

[5] Brandt AM (2007). The cigarette century.

[6] Borland R, Yong HH, Wilson N, Fong GT, Hammond D (2009). How reactions to cigarette packet health warnings influence quitting: Findings from the ITC four-country survey.. Addiction.

[7] Campaign for Tobacco-Free Kids (2009). FDA regulation of tobacco products: A common-sense plan to protect kids and save lives.. http://www.lungusa.org/atf/cf/%7B7a8d42c2-fcca-4604-8ade-7f5d5e762256%7D/FDA_BILL_SUMMARY.PDF.

[8] US Department of Health and Human Services (1989). Reducing the health consequences of smoking: 25 years of progress.. http://profiles.nlm.nih.gov/NN/B/B/X/S/.

[9] Siegel M, Carol J, Jordan J, Hobart R, Schoenmarklin S (1997). Preemption in tobacco control: Review of an emerging public health problem.. JAMA.

[10] Novotny TE, Zhao F (1999). Consumption and production waste: Another externality of tobacco use.. Tob Control.

[11] Barker KG (2009). Thank you for regulating: Why Philip Morris's embrace of FDA regulation helps the company but harms the agency.. Admin Law Rev.

[12] McDaniel PA, Malone RE (2005). Understanding Philip Morris's pursuit of US government regulation of tobacco.. Tob Control.

[13] McDaniel PA, Smith EA, Malone RE (2006). Philip Morris's Project Sunrise: Weakening tobacco control by working with it.. Tob Control.

[14] Berlind MH (1998). Design, manufacturing and marketing of tobacco products: Towards a sensible regulatory framework.. http://legacy.library.ucsf.edu/tid/lgs32a00.

[15] Goldman L, Glantz S (1994). Evaluation of antismoking advertising campaigns.. JAMA.

[16] Farrelly MC, Healton CG, Davis KC, Messeri P, Hersey JC (2002). Getting to the truth: Evaluating national tobacco countermarketing campaigns.. Am J Public Health.

[17] Farrelly MC, Davis KC, Haviland ML, Messeri P, Healton CG (2005). Evidence of a dose-response relationship between “truth” antismoking ads and youth smoking prevalence.. Am J Public Health.

[18] Farrelly MC, Nonnemaker J, Davis KC, Hussin A (2009). The influence of the national truth campaign on smoking initiation.. Am J Prev Med.

[19] Ling PM, Neilands TB, Glantz SA (2007). The effect of support for action against the tobacco industry on smoking among young adults.. Am J Pub Health.

[20] Ling PM, Neilands TB, Glantz SA (2009). Factors associated with young adult smoking behavior: A national survey.. Am J Prev Med.

[21] Giovino GA, Sidney S, Gfroerer JC, O'Malley PM, Allen JA (2004). Epidemiology of menthol cigarette use.. Nicotine Tob Res.

[22] Glantz SA, Barnes DE, Bero L, Hanauer P, Slade J (1996). The cigarette papers.

[23] United States District Court for the District of Columbia (2006). United States v. Philip Morris USA, Inc., et al. 449 F.Supp. 2d 1 (D.D.C. 2006).. http://library.ucsf.edu/tobacco/litigation/uspm.html.

[24] Barnes D, Bero LA (1996). Industry-funded research and conflict of interest: An analysis of research sponsored by the tobacco industry through the Center for Indoor Air Research.. J Health Polit Policy Law.

[25] Barnes D, Bero LA (1997). Scientific quality of original research articles on environmental tobacco smoke.. Tob Control.

[26] Barnes D, Bero LA (1998). Why review articles on the health effects of passive smoking reach different conclusions.. JAMA.

[27] Barnes D, Hanauer P, Slade J, Bero LA, Glantz S (1995). Environmental tobacco smoke. The Brown and Williamson documents.. JAMA.

[28] Barnoya J, Glantz S (2002). Tobacco industry success in preventing regulation of secondhand smoke in Latin America: The “Latin Project”.. Tob Control.

[29] Barnoya J, Glantz S (2005). The tobacco industry's worldwide ETS consultants project: European and Asian components.. Eur J Public Health.

[30] Bero L (2003). Implications of the tobacco industry documents for public health policy.. Annu Rev Public Health.

[31] Bero L (2005). Tobacco industry manipulation of research.. Public Health Rep.

[32] Bero L, Galbraith A, Rennie D (1994). Sponsored symposia on environmental tobacco smoke.. JAMA.

[33] Hirschhorn N, Bialous S (2001). Secondhand smoke and risk assessment: What was in it for the tobacco industry?. Tob Control.

[34] Hong M-K, Bero LA (2002). How the tobacco industry responded to an influential study of the health effects of secondhand smoke.. BMJ.

[35] Muggli ME, Forster J, Hurt RD, Repace J (2001). The smoke you don't see: Uncovering tobacco industry scientific strategies aimed against environmental tobacco smoke policies.. Am J Public Health.

[36] Ong E, Glantz S (2001). Constructing “sound science” and “good epidemiology”: Tobacco, lawyers, and public relations firms.. Am J Public Health.

[37] Schotland M, Bero LA (2002). Evaluating public commentary and scientific evidence submitted in the development of a risk assessment.. Risk Anal.

[38] United States Court of Appeals (2009). U.S.A. v. Philip Morris, et al. No. 06-5267.. http://pacer.cadc.uscourts.gov/docs/common/opinions/200905/06-5267-1181914.pdf.

[39] World Health Organization (2004). WHO framework convention on tobacco control.. http://www.who.int/fctc/en/index.html.

[40] Framework Convention on Tobacco Control Conference of the Parties (2008). Guidelines for implementation of Article 5.3: Guidelines on the protection of public health policies with respect to tobacco control from commercial and other vested interests.. http://www.who.int/fctc/guidelines/article_5_3/en/index.html.

[41] Blalock J, Burgard J (1963). Re: Tobacco Institute, Tobacco Industry Research Committee, and Hill & Knowlton.. http://legacy.library.ucsf.edu/tid/akc72d00.

[42] United States Supreme Court (1995). Rubin v. Coors Brewing Co., 514 U.S. 476.. http://caselaw.lp.findlaw.com/cgi-bin/getcase.pl?court=us&vol=514&invol=476.

[43] Szymanczyk M (2009). Letter to Henry Waxman endorsing Family Smoking Prevention and Tobacco Control Act.. http://energycommerce.house.gov/Press_111/20090304/hr1256_support.pdf.

